# Are differential consumption patterns in health-related behaviours an explanation for persistent and widening social inequalities in health in England?

**DOI:** 10.1186/s12939-016-0461-2

**Published:** 2016-10-18

**Authors:** Emma Stait, Michael Calnan

**Affiliations:** School of Social Policy, Sociology and Social Research, University of Kent, Canterbury, CT2 7NF UK

**Keywords:** Social inequalities in health, Health-related behaviour, England

## Abstract

**Background:**

During the last two decades, differential consumption patterns in health-related behaviours have increasingly been highlighted as playing an important role in explaining persistent and widening health inequalities. This period has also seen government public health policies in England place a greater emphasis on changing ‘lifestyle’ behaviours, in an attempt to tackle social inequalities in health. The aim of this study was to empirically examine the variation in health-related behaviour in relation to socio-economic position, in the English adult population, to determine the nature of this relationship and whether it has changed over time.

**Methods:**

The study population was derived from the Health Survey for England between 2001 and 2012 (*n* = 56,468). The relationships between health-related behaviour (smoking, fruit and vegetable intake, alcohol consumption and physical activity) and three socioeconomic indicators (educational level, occupational social class and equivilised household income) were analysed using log bi-nomial regression.

**Results:**

The study found that each of the three socio-economic indicators were statistically related to smoking, fruit and vegetable consumption and alcohol intake, with the strongest relationship found for smoking. For physical activity, no relationship was found in 2003 by education or income and in 2008 by occupation. Statistical analysis showed that the difference between those at the highest and lowest end of the socio-economic indicators had widened in relation to smoking, as measured by educational level, occupation and household income. A similar trend was also found for physical activity as measured by educational level and household income. However, for fruit and vegetable intake and alcohol consumption, the relationship between health-related behaviour and socio-economic position had narrowed over time as measured by education and income.

**Conclusions:**

The findings provided only partial support for the thesis that socio-economic variations in health-related behaviours may be significant in explaining widening health inequalities. The significance of socio-economic variations in health-related behaviours might reflect both materialist and cultural explanations for socio-economic inequalities although it was not possible to separate and estimate the relative importance of these effects.

**Electronic supplementary material:**

The online version of this article (doi:10.1186/s12939-016-0461-2) contains supplementary material, which is available to authorized users.

## Background

It is widely acknowledged that substantial differences in health status and longevity exist in England between the most advantaged and the least advantaged groups in society, with those higher up the social hierarchy generally enjoying better health and longevity than those lower down [1, 2]. These social inequalities in health, linked to socio-economic position (SEP), have been persistent in England (and indeed the United Kingdom (UK)) and in some areas have widened [3, 4] despite general improvements in health across the whole population.

A considerable literature exists which seeks to explain the relationship between SEP and health, with the publication of the Black Report in 1980, generally regarded as the catalyst for the health inequalities debate. Black came down strongly in favour of a materialist explanation, which highlighted the direct effects of material and structural factors in producing inequalities in health [5], although the report did identify the influence of cultural factors in explaining the variation in the take-up of health promotion and prevention programmes. Since the Black report, explanations for health inequalities have predominantly tended to focus on the direct and to an extent, indirect effects (e.g. stress and relative deprivation [6]) of material and structural factors. To an extent the cultural explanation has become conflated with materialist explanations [7, 8], although the explanatory power of such an approach has been contested by those who have pointed to the evidence that material factors, have a direct effect on health inequalities after allowing for the influence of health-related behaviour [9]. This conflated approach has been reflected, at least to some extent, in government policies in England following the Independent Review of Health Inequalities, which placed some emphasis on reducing absolute and relative disadvantage, through attempts to reduce poverty, improve material circumstances and address income inequalities in order to tackle health inequalities [10, 11]. This policy emphasis was short lived, as there was a noticeable shift towards a focus on changing consumption patterns in individual health-related behaviours following publication of the public health White Paper, “Choosing health” in 2004 [12]. These policies tended to focus on tackling smoking, excess alcohol consumption, poor diet and lack of physical activity, as these were the behaviours that were considered to be responsible for a large share of preventable mortality and morbidity in developed societies [13]. Despite this change in approach to addressing health inequalities, socio-economic differences in mortality rates continued to rise and the Marmot Review [14] into health inequalities in England, commissioned in 2008, reported that inequalities “cannot be attributed simply to genetic, bad, unhealthy behaviour or difficulties in access to health care” (p.16) but emphasized the interaction of material, social, behavioural, psychosocial and biological factors in generating socio-economic differences in heath.

In spite of Marmot’s recommendation that action be targeted across all social determinants of health, there was a continued and increasing emphasis on a predominantly behaviourist approach in health policy [15]. This approach may be linked to the increasing dominance of the neoliberal ideology in policy discourse with its emphasis on rational choice and individual responsibility [16]. These choices are generally considered to be under the control of the individual and consequently policies have placed an emphasis on health promotion strategies designed to support individuals to lead healthier lives and take responsibility for their health-related behaviours [12, 15]. This has manifested itself in recent English government policy which has promoted health and welfare (paternalism) [17] and at the same time as ensuring freedom of choice (libertarianism). A policy approach which has been exemplified by the behaviourist approach of ‘nudging’ people to make healthy choices by changing the architecture or landscape in which decisions are made.

Not only has the idea of health-related behaviour as underpinning inequalities in health, been manifest in policy but more recently some commentators have posited that socio-economic variations in health-related behaviours may be significant in explaining widening health inequalities in developed countries such as England. For example, Mackenbach [18] postulates that this may have occurred through the differential penetration of the diffusion of innovations, such as health promotion policies, with those in a higher SEP adopting new behaviours and taking up new interventions before those of a lower SEP. This might be combined with the current stage of epidemiological transition where health improvement depends largely on behavioural changes [19]. Thus, non-material factors for health such as cultural capital may have an increased importance. However, this cultural explanation differs from the one proposed in the Black report in that inequalities in health-related behaviours between socio-economic groups are seen to be a product of the need for ‘social distinction’ due to the reduction in opportunities to distinguish between groups on the basis of visible signs of material prosperity [20, 21]. This also might reflect more general social economic changes and a shift from Fordist principles to post-Fordism with the emphasis on social identity being tied to position in the consumption rather than the production process [19].

This paper explores the power of the consumption explanation through an empirical analysis of the relationship between SEP and health-related behaviours in England by investigating the nature of this relationship and whether it has widened over time and if the evidence showed consistent differences in health-related behaviours between different socio-economic groups. The analysis focused on the consumption patterns of four health-related behaviours in English adults which were smoking, excessive alcohol consumption, low fruit and vegetable intake and lack of physical activity as these are the health-related behaviours that the government policies have targeted in their public health policies to reduce health inequalities in England [12].

## Methods

### Study population

The study population was derived from the Health Survey for England (HSE), which is an annual cross-sectional survey designed to collect information from a nationally representative sample of those aged 16 years and over, living in private households in England [22–27]. A description of the methods used in the HSE are detailed elsewhere [28]. The HSE collects a variety of demographic, socioeconomic and health data using questionnaires and objective measures of health. A two-stage stratified random sampling process is used with the Postcode Address File as the primary sampling unit. Individuals selected for inclusion in one survey year are excluded from selection during the following 3 years [28]. Estimated adult interview response rates have declined since the survey was introduced from approximately 70 % in the 1990s to approximately 60 % in the 2000s [28]. The total dataset for selected survey years consisted of 76,628 respondents. However, for this study, only data on respondents aged 18–64 years were analyzed. Respondents aged 65 years and over were excluded as socio-economic indicators for this population can present methodological problems e.g. only a small proportion over 65 years work and many of the current older population have few or no academic qualifications. Respondents aged 16–17 years were also excluded, as were respondents with missing data on any ‘lifestyle’ and socio-economic factors. The final dataset consisted of 56,468 respondents.

### Variables

#### Health-related behaviours

Binary variables were created for the four health-related behaviours (smoking, alcohol consumption, fruit and vegetable intake and physical activity) and benchmarked against current UK guidelines to determine whether respondents had ‘met’ or ‘not met’ national recommendations. For smoking, respondents were asked whether they smoked cigarettes nowadays. Those answering ‘yes’ were classified as smokers and those answering ‘no’ were classified as non-smokers. Former smokers were classified as non-smokers, regardless of the length of time since stopping. Alcohol consumption was based on the amount of alcohol (in units) consumed on the heaviest day during the previous 7 days. National guidelines for daily alcohol consumption at the time were no more than 3–4 units for men and 2–3 units for women [29], therefore for this study, excessive alcohol consumption was defined as an intake of >4 units for men and >3 units for women on the heaviest drinking day. Since 2006, a number of changes have been made to the way HSE estimates alcohol consumption therefore conversion factors were applied to ensure the data was comparable across time. A description of these changes are detailed elsewhere [30]. Fruit and vegetable consumption was calculated by adding up the number of ‘portions’ of fruit and vegetables eaten the day before the interview and included pulses, salad, vegetables, fruit and fruit juice. Low fruit and vegetable consumption was defined as less than the recommended five portions of fruit and vegetables a day [31]. The current physical activity recommendation for adults is at least 150 min of moderate intensity activity each week (including occupational activity, heavy housework, gardening, walking and sports), which may be achieved by doing 30 min on at least 5 days a week [32]. A number of changes have been made to physical activity guidelines since 2004, therefore to ensure comparability of the data, physical activity was calculated by the number of days each week of moderate physical activity for 30 min or more during the previous 4 weeks. Lack of physical activity was defined as less than 20 days at moderate intensity during the last 4 weeks.

#### Socio-economic indicators

It is widely acknowledged that SEP is formed along a range of different dimensions and that different socio-economic indicators measure different aspects of social stratification [33]. This study used three different indicators of SEP in order to better capture the potentially differing underlying mechanisms influencing health-related behaviour. Education is a strong determinant of access to employment opportunities and income and therefore reflects material resources [34], with higher educational levels more likely to be associated with healthier lifestyles. Education is also likely to reflect cognitive skills and knowledge, which may make people more receptive to health messages and modification of risky health behaviours [33]. However, it has also been suggested that the link between education and lifestyle might be influenced by the cultural context in which people are raised as some research suggests that health-related behaviours are associated with childhood SEP [35]. Occupation is widely used as a measure of SEP in the UK and is strongly related to income, which is an indication of material resources. It is also related to social standing and is thought to reflect psychosocial links connected to stress, control, autonomy and social integration [33]. It has been suggested that differences in health-related behaviour between groups of differing social status might also be a reflection of shared culture or lifestyle, which influences the health-related behaviours considered to be appropriate to a group [36]. Income is the SEP indicator that most directly measures material resources. Both educational and occupational indicators measure different aspects of SEP and although it is not possible to separate these aspects, it is suggested that they may be identifying a mix of both materialist and cultural influences. Income however, should reflect more directly, material influences on health-related behaviour.

Educational level was measured using the highest educational qualification obtained and compared higher education (degree or equivalent) and no qualifications. Occupational social class was based on the 3-class version of the National Statistics Socio-Economic Classification (NS-SEC). The NS-Sec was introduced in 2001 and classifies groups on the basis of employment relations and conditions such as mode of payment, promotion prospects and levels of autonomy [37]. Occupation was based on the current or former occupation of the household reference person and compared professional and managerial groups and routine and manual groups. Income was measured by annual equivalised household income, which is calculated by adjusting the total annual household income to account for different needs of different size households. Income was divided into five groups and the highest and lowest fifths were compared.

#### Demographic variables

The demographic variables of gender and age were included in the analysis as possible confounding factors. Age was categorized into five bands: 18–24, 25–34, 35–44, 45–54 and 55–64.

### Analyses

Due to the omission in some survey years of questions relating to lifestyle indicators, it was not possible to use the same surveys for analysis of health-related behaviours. Therefore 2001, 2006 and 2011 survey data were analysed for smoking, fruit and vegetable intake and alcohol consumption and 2003, 2008 and 2012 survey data for physical activity. Prevalence rates (%) for the four health-related behaviours were determined for each socio-economic indicator to give an indication of the nature of the relationship. Prevalence rate ratios were estimated by log binomial regression to determine the strength of the relationship and relative differences between each SEP indicator and health-related behaviour (adjusting for gender and age) to see whether this relationship had changed over time. The differences were expressed as relative risk ratios, which represented the risk of smoking and not meeting national recommendations for fruit and vegetable intake, alcohol consumption and physical activity in the lowest socio-economic groups as compared to the highest socioeconomic groups. In order to determine whether the relationship between SEP and each health-related behaviour differed significantly between survey years, interaction tests were conducted using a combined dataset. Survey weights were incorporated into the analyses, with the exception of 2001 survey data, as weights were not provided. All analyses conducted looked at health-related behaviours across different SEPs but as the focus of the study was on the gap between the highest and lowest socio-economic groups, it is just these figures that are reported in the findings. All analyses were performed using SPSS version 22.0.

## Results

**Fig. 1:**
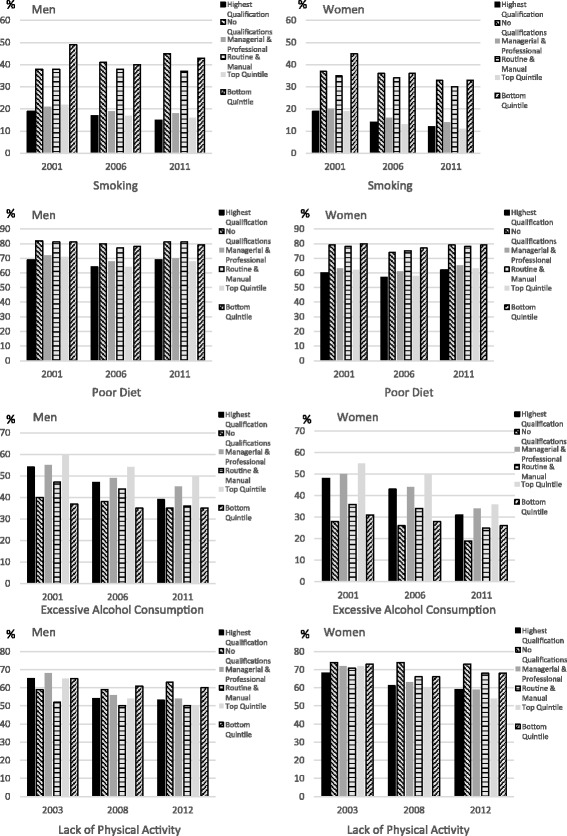
Observed prevalence rates (%) for health-related behaviour by educational level, occupation, income and gender

Observed prevalence rates for each health-related behaviour for men and women by the lowest and highest indicators for education, occupation and income for each survey year are shown in Fig. 1, while observed prevalence rates for each health-related behaviour for each age category by the lowest and highest indicators for education, occupation and income are given in Tables S1–S4 (see Additional file 1: Tables S1–S4). Unless otherwise stated the findings discussed relate to all three survey years for each health-related behaviour.

A clear inverse relationship was found between smoking and all socio-economic indicators, for both men and women and for all age categories, with smoking prevalence highest among the lowest socio-economic groups compared to the highest socio-economic groups. Overall, smoking was more common among men than among women. In terms of age, smoking prevalence was highest among 18–24 year olds and lowest among 55–64 year olds for all SEP indicators, except for those in the bottom income group in 2011 when smoking prevalence was higher among 55–64 year olds compared to 18–24 year olds.

Daily fruit and vegetable consumption showed a positive relationship with the lowest intake among men and women in the lowest socio-economic groups compared to the highest socio-economic groups. The same relationship was found across all age groups in relation to SEP except for 18–24 year olds in the highest and lowest income groups in 2006 and 2011 and the highest and lowest occupational groups in 2011, when fruit and vegetable consumption was the same. In general, daily fruit and vegetable intake was proportionately lower for men than for women and for the youngest age group compared to the oldest age group.

Daily alcohol consumption was greatest among the highest socio-economic groups for men and women and across all age categories, while overall daily consumption was greatest among men compared to women and for 18–24 years olds compared to 55–64 years olds, except in 2011 when alcohol consumption was slightly higher for the oldest group with no qualifications compared to the youngest group with no qualifications.

The relationship between physical activity and SEP was more mixed with physical activity levels found to be lowest among men and women in the lowest socio-economic groups, except for 2003, when it was lowest among men and women in the highest occupational group and for men in the highest educational group, and in 2008 and 2012 when it was lowest among men in the highest groups as measured by occupation. In terms of age, the relationship between physical activity and SEP varied (see Additional file 1: Tables S1–S4). In 2003 physical activity tended to be lowest for more age groups in the highest educational and occupational groups but in 2008 and 2012 it tended to be lowest among the lowest educational and income groups for most ages. More generally physical activity was lower for women than for men and for 55–64 year olds compared to 18–24 year olds.

**Fig. 2:**
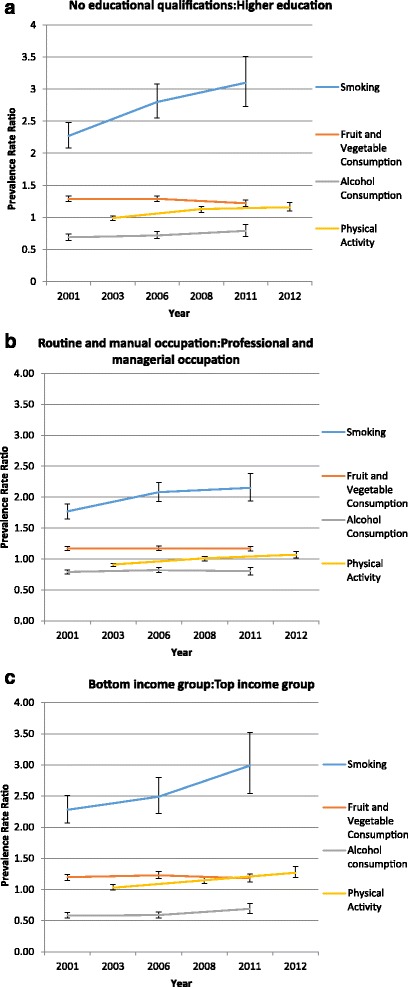
Changes over time for the relationship between SEP and health-related behaviour for selected survey years. Notes: The values on the vertical axis give the relative risk (PR) of a respondent in the lowest versus the highest socio-economic group, engaging in a particular health-related behaviour (adjusting for gender). Reference groups for predictor variables are higher education, managerial and professional and highest income group. Reference groups for outcome variables are non-smoker, ≥5 portions of fruit and vegetables daily, ≤ 4 units (men) and ≤3 units (women) per day for alcohol consumption, ≥20 days of moderate physical activity for 30 min or more during the last 4 weeks

Figure 2 shows the strength of the relationship and the relative differences for each health-related behaviour and socio-economic indicator for all survey years as well as the changes in prevalence rate ratios over time. The values on the vertical axis give the prevalence rate ratios of a respondent in the lowest versus the highest SEP engaging in a particular health-related behaviour (adjusting for gender and age) (Additional file 2: Tables S5–S7 shows the prevalence rate ratios and confidence intervals in more detail). In terms of the strength of the relationship between the four health-related behaviours and each SEP indicator, the data suggests that the association was strongest for smoking for all three socio-economic indicators. Over time the relationships were found to strengthen for smoking as measured by all three socio-economic indicators and for physical activity as measured by education and income. However, the relationship appears to have weakened for alcohol consumption as measured by all three socio-economic indicators and for diet as measured by education and income.

*Educational level*: In 2001, those with no qualifications had a higher risk of smoking (PR = 2.27; CI = 2.08–2.48) and not meeting daily recommendations for fruit and vegetable intake (PR = 1.29; CI = 1.25–1.33) compared to those with a higher education. By 2011 the risk of smoking had increased for those with no qualifications (PR = 3.10; CI = 2.73–3.51) resulting in a widening of the smoking gap between the highest and lowest educational groups. However, fruit and vegetable intake had decreased by 2011 for those with no qualifications (PR = 1.22; CI = 1.17–1.27), therefore the gap between the highest and lowest educational groups for fruit and vegetable intake narrowed over time (see Fig. 2a). Interaction tests showed that the changes over time for both smoking and fruit and vegetable consumption were statistically significant between 2001 and 2011 (*p* = 0.003 and *p* = 0.018 respectively) but not between 2001 and 2006. Daily alcohol consumption showed a different pattern in 2001 with the no qualifications group having a lower risk of exceeding alcohol consumption guidelines (PR = 0.69; CI = 0.64–0.74) compared to the higher education group. By 2011 the risk of exceeding alcohol consumption guidelines had increased slightly for those with no qualifications (PR = 0.79; CI = 0.70–0.89), which saw the gap narrow across time. Interaction tests showed that the changes over time were not statistically significant. No significant differences were found in levels of physical activity in 2003 between the highest and lowest socio-economic groups as measured by education, however between 2008 and 2012 those with no qualifications had a higher risk of not meeting physical activity guidelines (PR = 1.13; CI = 1.08–1.17 and PR = 1.16; CI = 1.10–1.23, respectively) compared to those with a higher education. Figure 2a shows that between 2003 and 2012 the gap for physical activity had widened and interaction tests showed that these changes were statistically significant between 2003 and 2008 (*p* = 0.000) and between 2003 and 2012 (*p* = 0.000).

*Occupational social class*: In 2001, those in routine and manual occupations had a higher risk of smoking (PR = 1.77; CI = 1.65–1.89) and having a poorer diet (PR = 1.17; CI = 1.14–1.20) compared to those in managerial and professional occupations. By 2011 the risk of smoking for those in the lowest occupational group had increased (PR = 2.15; CI = 1.94–2.38) and therefore the gap for smoking widened between the highest and lowest occupational groups. However the gap for fruit and vegetable intake remained the same across the period (see Fig. 2b). Interaction tests showed that the changes over time were only statistically significant for smoking between 2001 and 2011 (*p* = 0.047). Again daily alcohol consumption showed a different socio-economic patterning in 2001 with the routine and manual group having a lower risk of drinking heavily (PR = 0.79; CI = 0.76–0.82) compared to the managerial and professional group, although by 2011, alcohol consumption for the routine and manual group had changed very little (PR = 0.80; CI = 0.74–0.86) from 2001. Interaction tests showed that the changes over time for daily alcohol intake were not statistically significant. In 2003, routine and manual workers were found to have a lower risk of meeting physical activity guidelines (PR = 0.91; CI = 0.88–0.93) compared to managerial and professional workers. By 2011, the risk of not meeting physical activity guidelines had increased for routine and manual workers (PR = 1.07; CI = 1.02–1.12) compared to managerial and professional workers and although the gap had narrowed between these groups by 2008, it had widened by 2011. Interaction tests showed that the changes over time to physical activity levels between the highest and lowest occupational groups were statistically significant between 2003 and 2008 (*p* = 0.005) and between 2008 and 2012 (*p* = 0.000).

*Household income*: In 2001, the risk of being a smoker and having a low intake of fruit and vegetables was highest for lowest income households (PR = 2.28 CI = 2.07–2.51 and PR = 1.20; CI = 1.15–1.24, respectively) compared to highest income households. By 2011 the risk of smoking had increased (PR = 2.99; CI = 2.54–3.52) and the consumption of fruit and vegetables had decreased (PR = 1.18; CI = 1.12–1.25) for lowest income households. Consequently, the gap for smoking widened across the period whereas the gap for fruit and vegetable consumption narrowed slightly (see Fig. 2c). Interaction tests showed that only the change over time for smoking between 2001 and 2011 was statistically significant (*p* = 0.030). Conversely daily alcohol consumption in 2001 was lowest among least affluent households (PR = 0.58; CI = 0.54–0.63) compared to the most affluent households, although by 2011, alcohol consumption had increased for those in the lowest income households (PR = 0.69; CI = 0.62–0.78). This suggests that the relationship between the highest and lowest income groups narrowed between 2001 and 2011. Interaction tests showed that the changes over time for alcohol consumption were statistically significant between 2001 and 2011 (*p* = 0.019) but not between 2001 and 2006. For physical activity no significant difference was found in meeting recommendations in 2003 between the lowest and highest income groups, however from 2008 to 2012, the risk of not meeting guidelines was higher for lowest income households (PR = 1.15; CI = 1.10–1.21 and PR = 1.27; CI = 1.19–1.37 respectively) compared to highest income households. Between 2003 and 2012 the gap for physical activity levels appeared to widen and interaction tests showed that the change over time in physical activity was statistically significant between 2003 and 2012 (*p* = 0.000) but not between 2003 and 2008.

## Discussion

Strong support for the explanatory power of the consumption thesis for social inequalities in health would show a widening of the relationship between the highest and lowest socio-economic indicators for the four different health-related behaviours across the period of analysis. The results provided only partial support for the consumption argument with the strongest supportive evidence showing that between 2001 and 2012, the gap between the highest and lowest socio-economic indicators widened for smoking as measured by education, occupation and income. There was also some support for the thesis in relation to physical activity where the gap appeared to widen for education and income and for alcohol consumption where the gap appeared to narrow for these two indicators, although it must be remembered that alcohol consumption is more likely to be positively related to socio-economic position. However, there appeared to be no support for the thesis in relation to fruit and vegetable consumption as a narrowing gap over time was found when measured by education and to a much lesser extent by income, but no change when measured by occupation. In terms of the strength of the relationship between each health-related behaviour and SEP indicator, the data suggests that these relationships were found to strengthen over time for smoking and physical activity on all three indicators. For alcohol consumption and diet the relationship appears to have weakened as measured by education and income.

The partial support for the consumption thesis might be a reflection on the limitations of the study. Self-reporting of health-related behaviour has the potential for reporting bias with the under-reporting of smoking and alcohol consumption and over-reporting of fruit and vegetable consumption and physical activity due to social-desirability. The variability in the pattern of the relationship between socio-economic position and changes in health-related behaviours over time might therefore be explained by the latter being based on self-reports. Certainly, it has been argued that smoking has become increasingly stigmatised, which has obvious implications for disclosure in reporting [38]. It may also be difficult to generalize the findings of this study to other populations as the health measures and cut-off points used were in accordance with current UK guidelines. Also by making the alcohol data comparable in this study, there is a possibility that alcohol consumption may have been underestimated although by looking at trends over time, this impact may be reduced. However, the period of analysis coincided with an increasing emphasis on lifestyle change policies in England [12] and as the HSE is specifically designed to collect information on health and health-related behaviours in relation to certain socio-economic characteristics to inform policies, the data was considered appropriate to address the research questions. The extent to which these findings are generalizable to other countries may also be problematic given the arguments that the strength and nature of the relationship between socio-economic position and health-related behaviours is associated with the structure and nature of health systems and welfare regimes [39].

One other limitation of the study was in the operationalisation of the concept of culture. The aim was to shed light on the explanatory power of the materialist compared with the cultural explanation of social inequalities in patterns of health-related behaviour. The evidence suggested that patterns in the strength of the relationship between the health-related behaviours appear to be strongest as measured by education and then income but weakest as measured by occupation. Thus, if, as has been argued, educational qualifications can be used as proxy indicator of cultural position then the evidence suggests that non-material influences might be important explanatory influences along with material circumstances. This needs to be explored in more detail in studies using mixed methods where concepts of cultural position and perspective are more precisely operationalized rather than using proxy indicators such as educational qualifications as in this study. There are a number of competing cultural explanations which need to be explored in this further research. For example, it has been suggested that while cultural attitudes may not be salient in explaining differences in patterns of health-related behaviour, the concept of habitus may be more important where these behaviours have become taken for granted and routinized in the form of habits which are passed on from generation to generation, possibly through education [36]. Alternative explanations, also based on concepts derived from Bourdieu’s theory, suggest that health-related behaviours might be a way in which social groups are able to express and reinforce ‘social distinction’ from other groups [20], where smoking might be seen to be a legitimate indicator of social identity reflected in socio-economic position. However, fruit and vegetable intake may not be an appropriate indicator in terms of explaining socio-economic inequalities in health and other ‘diet’-related factors may be a more relevant measure. A further alternative explanation, closely related to culture, emphasizes the importance of status-based differences in the adoption and diffusion of innovative behaviours that emphasize the importance of symbolic boundaries, partly defined by the innovative behaviour (e.g. smoking) of high socio-economic groups and the lag in the adoption of these behaviours by those in low socio-economic groups [40]. Thus, this might also suggest that the recent health promotion policies aimed at behavioural and lifestyle change adopted in England by the government might be more effective at influencing the practices of the advantaged compared with disadvantaged groups [38] and thus have the effect of creating further inequalities.

Finally, the analysis shed some light on the question of the relationship between health-related behaviours and socio-economic position and whether it is best explained by typifying the behaviours as independent or as co-occurring and/or as clusters. Certainly, there is evidence of co-occurrence and/or clustering of unhealthy behaviours among those in lower SEP’s (e.g. [41–43]) although there are major methodological challenges associated with exploring these relationships [44] which may make it difficult to compare findings between studies due to the different conceptualizations and analyses used. Yet it is also evident from the data in this study that the socio-economic indicators are differentially related to the health-related behaviours. This might suggest that the conceptualisation of lifestyle in terms of a unified cluster of health-related behaviours associated with socio-economic position might have limited explanatory power at least in terms of widening social inequalities in health [45]. The findings from this study appears to suggest that some so-called lifestyle behaviours might be more important for explaining social inequalities in health than others. The evidence clearly points to the salience of smoking and although the findings might suggest a materialist explanation, particularly in relation to the strong relationship found between smoking and income, it does not explain why those with the least money are more likely to smoke and smoke the most [46, 47], which might reflect the indirect effects of coping with stress from relative deprivation or once again points to the importance of the interrelationship with cultural influences.

## Conclusion

The findings appeared to show both a widening and a narrowing of consumption patterns in relation to socio-economic position suggesting that the consumption thesis may have some, if only limited, explanatory power. Therefore, it is necessary to look for other explanations for the increase in health inequalities, including the direct effect of material circumstances and psychosocial factors [18]. In terms of explanations for the variation in consumption patterns for health-related behaviours it is suggested that the findings reflect both material and cultural influences although it was not possible to separate and estimate the relative importance of these effects due to the lack of precise indicators of cultural position. Thus, there is a need to further explore the extent to which social inequalities in health-related behaviour reflect cultural as well as materialist explanations. Further research should also examine the relationship between the changing trends in these consumption patterns and health outcomes and longevity. Current English government policy tends to portray individual health-related behaviours as independent and there is some evidence to support such an approach from this analysis, which showed that the socio-economic indicators were differentially related to the different health-related behaviours. An alternative or complimentary policy approach might focus on multiple health-related behaviours although further evidence needs to be gathered about the relative importance of the extent to which health-related behaviours co-occur and/or cluster in their relationship with SEP and how these change over time.

## Additional files

Additional file 1: Tables S1–S4. Observed prevalence rates (%) for health-related behaviour for age categories by educational level, occupation and income. (DOCX 21 kb)
Additional file 2: Tables S5–S7. Prevalence rate ratios for health-related behaviour by SEP indicators. (DOCX 17 kb)

## Abbreviations

CI: Confidence interval
HSE: Health Survey for England
NS-Sec: National Statistics Socio-economic Classification
PR: Prevalence rate
SEP: Socio-economic position
UK: United Kingdom
