# Supplementary material for: Are differential consumption patterns in health-related behaviours an explanation for persistent and widening social inequalities in health in England?
Source: Int J Equity Health. 2016 Oct 18;15:171. doi: 10.1186/s12939-016-0461-2 (PMC5070153; doi:10.1186/s12939-016-0461-2)
Supplement: Additional file 1: Tables S1–S4. — Observed prevalence rates (%) for health-related behaviour for age categories by educational level, occupation and income. (DOCX 21 kb) [file 12939_2016_461_MOESM1_ESM.docx]

Tables S1-S4 Observed prevalence rates (%) for health-related behaviour for age categories by educational level, occupation and income.

Table S1

Smoking prevalence (%)

| Year |  | Educational level | | Occupation | | Income | |
| --- | --- | --- | --- | --- | --- | --- | --- |
|  |  | Highest | No Qualific-  ations | Managerial & Professional | Routine & Manual | Top Quintile | Bottom Quintile |
| 2001 | 18-24  25-34  35-44  45-54  55-64 | 30  24  19  16  11 | 64  48  47  36  27 | 26  26  19  20  13 | 44  41  38  33  28 | 27  24  21  19  11 | 50  51  51  43  33 |
| 2006 | 18-24  25-34  35-44  45-54  55-64 | 19  18  17  14  8 | 52  49  40  37  32 | 26  21  17  16  11 | 32  41  38  36  31 | 27  15  17  15  10 | 35  47  38  37  32 |
| 2011 | 18-24  25-34  35-44  45-54  55-64 | 16  16  14  14  8 | 50  40  39  46  32 | 22  18  15  15  12 | 30  35  38  36  27 | 20  15  12  13  11 | 28  44  42  38  36 |

Table S2

Fruit and vegetable consumption (%) (less than 5 portions daily)

| Year |  | Educational level | | Occupation | | Income | |
| --- | --- | --- | --- | --- | --- | --- | --- |
|  |  | Highest | No Qualific-ations | Managerial & Professional | Routine & Manual | Top Quintile | Bottom Quintile |
| 2001 | 18-24  25-34  35-44  45-54  55-64 | 72  71  65  60  57 | 89  86  86  78  76 | 79  72  68  63  60 | 84  82  81  76  74 | 74  71  69  63  58 | 81  84  84  81  72 |
| 2006 | 18-24  25-34  35-44  45-54  55-64 | 76  64  61  60  52 | 84  75  76  81  75 | 77  68  65  63  56 | 83  75  77  75  74 | 82  62  65  55  54 | 82  76  74  80  75 |
| 2011 | 18-24  25-34  35-44  45-54  55-64 | 76  70  65  66  57 | 91  80  78  85  77 | 83  72  67  66  58 | 83  76  81  82  78 | 75  72  65  67  54 | 75  85  83  79  74 |

Table S3

Alcohol consumption (%) (more than 4 units (men) and 3 units (women) per day

| Year |  | Educational level | | Occupation | | Income | |
| --- | --- | --- | --- | --- | --- | --- | --- |
|  |  | Highest | No Qualific-ations | Managerial & Professional | Routine & Manual | Top Quintile | Bottom Quintile |
| 2001 | 18-24  25-34  35-44  45-54  55-64 | 69  53  52  48  45 | 33  31  36  37  29 | 65  54  52  50  48 | 53  44  43  41  29 | 67  60  58  55  55 | 46  36  32  30  22 |
| 2006 | 18-24  25-34  35-44  45-54  55-64 | 54  46  43  45  44 | 30  33  32  33  31 | 56  49  44  46  44 | 42  41  41  39  31 | 51  55  51  50  50 | 38  33  29  28  27 |
| 2011 | 18-24  25-34  35-44  45-54  55-64 | 38  37  35  33  36 | 25  25  26  32  27 | 46  43  40  37  33 | 38  25  33  31  29 | 52  47  42  41  43 | 38  29  25  32  26 |

Table S4

Physical activity (%) (less than 30 minutes of moderate physical activity on 20 days during the last four weeks)

| Year |  | Educational level | | Occupation | | Income | |
| --- | --- | --- | --- | --- | --- | --- | --- |
|  |  | Highest | No Qualific-ations | Managerial & Professional | Routine & Manual | Top Quintile | Bottom Quintile |
| 2003 | 18-24  25-34  35-44  45-54  55-64 | 52  65  68  69  70 | 57  63  62  62  74 | 59  68  70  73  75 | 59  60  59  60  71 | 56  67  70  68  70 | 58  70  66  73  84 |
| 2008 | 18-24  25-34  35-44  45-54  55-64 | 46  54  57  61  63 | 50  53  63  69  75 | 48  55  60  62  66 | 51  50  54  61  74 | 50  54  53  59  64 | 47  58  65  72  82 |
| 2012 | 18-24  25-34  35-44  45-54  55-64 | 48  55  54  56  64 | 60  58  66  66  75 | 45  55  56  58  65 | 58  52  55  60  71 | 34  50  53  52  60 | 53  58  63  71  80 |
