# Supplementary material for: Are differential consumption patterns in health-related behaviours an explanation for persistent and widening social inequalities in health in England?
Source: Int J Equity Health. 2016 Oct 18;15:171. doi: 10.1186/s12939-016-0461-2 (PMC5070153; doi:10.1186/s12939-016-0461-2)
Supplement: Additional file 2: Tables S5–S7. — Prevalence rate ratios for health-related behaviour by SEP indicators. (DOCX 17 kb) [file 12939_2016_461_MOESM2_ESM.docx]

Tables S5-S7 Prevalence rate ratios for health-related behaviour by SEP indicators.

Table S5

Prevalence ratios (PR) of health-related behaviour by educational level

|  |  | 2001/2003* | | 2006/2008* | 2011/2012* | |
| --- | --- | --- | --- | --- | --- | --- |
| Health-related behaviour | Educational level | PR  95% CI |  | PR  95% CI |  | PR  95% CI |
| Smoker | Highest | 1.00 |  | 1.00 |  | 1.00 |
|  | No qualifications | 2.27  2.08-2.48 |  | 2.80  2.55-3.08 |  | 3.10  2.73-3.51 |
| Daily fruit and vegetable consumption (<5 portions) | Highest | 1.00 |  | 1.00 |  | 1.00 |
|  | No qualifications | 1.29  1.25-1.33 |  | 1.29  1.25-1.33 |  | 1.22  1.17-1.27 |
| Daily alcohol consumption (>4 units for men, >3 units for women) | Highest | 1.00 |  | 1.00 |  | 1.00 |
|  | No qualifications | 0.69  0.64-0.74 |  | 0.72  0.67-0.78 |  | 0.79  0.70-0.89 |
| Physical activity (< 20 days of moderate physical activity for 30 minutes or more during the last four weeks) | Highest | 1.00 |  | 1.00 |  | 1.00 |
|  | No qualifications | 0.99  0.95-1.02 |  | 1.13  1.08-1.17 |  | 1.16  1.10-1.23 |

Table S6

Prevalence ratios (PR) of health-related behaviour by occupational social class

|  |  | 2001/2003* | | 2006/2008* | 2011/2012* | |
| --- | --- | --- | --- | --- | --- | --- |
| Health-related behaviour | Occupational Social Class | PR  95% CI |  | PR  95% CI |  | PR  95% CI |
| Smoker | Managerial and Professional | 1.00 |  | 1.00 |  | 1.00 |
|  | Routine and Manual | 1.77  1.65-1.89 |  | 2.08  1.93-2.24 |  | 2.15  1.94-2.38 |
| Daily fruit and vegetable consumption (<5 portions) | Managerial and Professional | 1.00 |  | 1.00 |  | 1.00 |
|  | Routine and Manual | 1.17  1.14-1.20 |  | 1.17  1.14-1.21 |  | 1.17  1.13-1.20 |
| Daily alcohol consumption (>4 units for men, >3 units for women) | Managerial and Professional | 1.00 |  | 1.00 |  | 1.00 |
|  | Routine and Manual | 0.79  0.76-0.82 |  | 0.82  0.78-0.86 |  | 0.80  0.74-0.86 |
| Physical activity (< 20 days of moderate physical activity for 30 minutes or more during the last four weeks) | Managerial and Professional | 1.00 |  | 1.00 |  | 1.00 |
|  | Routine and Manual | 0.91  0.88-0.93 |  | 1.01  0.97-1.04 |  | 1.07  1.02-1.12 |

Table S7

Prevalence ratios (PR) of health-related behaviour by equivilised household income

|  |  | 2001/2003* | | 2006/2008* | 2011/2012* | |
| --- | --- | --- | --- | --- | --- | --- |
| Health-related behaviour | Household Income | PR  95% CI |  | PR  95% CI |  | PR  95% CI |
| Smoker | Top Quintile | 1.00 |  | 1.00 |  | 1.00 |
|  | Bottom Quintile | 2.28  2.07-2.51 |  | 2.49  2.22-2.80 |  | 2.99  2.54-3.52 |
| Daily fruit and vegetable consumption (<5 portions) | Top Quintile | 1.00 |  | 1.00 |  | 1.00 |
|  | Lowest Quintile | 1.20  1.15-1.24 |  | 1.23  1.18-1.29 |  | 1.18  1.12-1.25 |
| Daily alcohol consumption (>4 units for men, >3 units for women) | Top Quintile | 1.00 |  | 1.00 |  | 1.00 |
|  | Bottom Quintile | 0.58  0.54-0.63 |  | 0.59  0.54-0.64 |  | 0.69  0.62-0.78 |
| Physical activity (< 20 days of moderate physical activity for 30 minutes or more during the last four weeks) | Top Quintile | 1.00 |  | 1.00 |  | 1.00 |
|  | Bottom Quintile | 1.03  0.99-1.08 |  | 1.15  1.10-1.21 |  | 1.27  1.19-1.37 |

Reference groups for outcome variables are non-smoker, ≥5 portions of fruit and vegetables daily, ≤ 4 units (men) and ≤ 3 units (women) per day for alcohol consumption, ≥20 days of moderate physical activity for 30 minutes or more during the last four weeks.

*Survey years for 2001, 2006, 2011 were analysed for smoking, diet and alcohol consumption. For physical activity 2003, 2008 and 2012 were analysed.
